# Assessing the Diagnostic Value of Plasma-Free DNA in Prostate Cancer Screening

**DOI:** 10.29252/ibj.22.5.331

**Published:** 2018-09

**Authors:** Seyedeh Maryam Seyedolmohadessin, Mohammad Taghi Akbari, Zahra Nourmohammadi, Abbas Basiri, Gholamreza Pourmand

**Affiliations:** 1 Department of Biology, Science and Research Branch, Islamic Azad University, Tehran, Iran; 2 Department of Medical Genetics, Faculty of Medical Sciences, Tarbiat Modares University, Tehran, Iran; 3 Urology and Nephrology Research Center, Shahid Beheshti University of Medical Sciences, Tehran, Iran; 4 Urology Research Center, Tehran University of Medical Sciences, Tehran, Iran

**Keywords:** Cell-free DNA, Non-invasive diagnosis, Prostate cancer

## Abstract

**Background:**

Prostate cancer is the second form of cancer among men worldwide. For early cancer detection, we should identify tumors in initial stages before the physical signs become visible. The present study aims to evaluate the diagnostic value of cell-free DNA (cfDNA), its comparison with prostate-specific antigen (PSA) level in prostate cancer screening and also in patients with localized prostate cancer, metastatic form, and benign prostatic hyperplasia (BPH).

**Methods:**

The participants of this study were selected from 126 patients with genitourinary symptoms suspected prostate cancer, rising PSA, and/or abnormal rectal examination results and 10 healthy subjects as controls. Peripheral blood plasma before any treatment measures was considered. cfDNA was extracted using a commercial kit, and PSA levels were measured by ELISA. The ANOVA test was used to compare the average serum level of PSA and plasma concentration of cfDNA between the groups. The correlation between variables was measured by the Pearson test.

**Results:**

The subgroups consisted of 50 patients with localized prostate cancer, 26 patients with metastatic prostate cancer, 50 patients with BPH, and 10 healthy subjects; the average concentrations of cfDNA in these subgroups were 15.04, 19.62, 9.51, and 8.7 ng/μl, respectively. According to p < 0.0001 obtained from multivariate test, there was a significant difference between all the groups.

**Conclusion:**

Our findings indicated significant differences between cfDNA levels of patients with localized and metastatic prostate cancer, and differences of these two groups from BPH and healthy cases show the importance of this biomarker in non-invasive diagnostic procedures.

## INTRODUCTION

Prostate cancer is the second most common malignancy among men worldwide[1]. Iran holds the first rank in terms of the incidence of prostate cancer among Asian countries[2], and this type of cancer has been reported as the second most common cancer after gastric cancer among men in Tehran[3].

Recognition of tumors in the early stages contributes to the rapid detection of cancer before physical manifestation of the disease. In such stages, identifying the smallest tumors can be possible by detecting their specific tracks (biomarkers) in the bloodstream[4]. Biomarkers can indicate a normal physiological state or a disease state such as progression or response to treatment[5]. The prostate-specific antigen (PSA) has been introduced as a routine biomarker for the early detection of prostate cancer[6]. However, it has recently been shown that PSA is not a reliable diagnostic test[7]. The main concern surrounding the use of this marker for prostate cancer screening is the lack of specificity. An increase in the levels of PSA can reflect the presence of cancer cells, but it may also represent non-malignant states such as benign prostatic hyperplasia (BPH), infection, or chronic inflammation[8]. Given these issues and changes in the relationship between total PSA and the stage of disease[9], it seems that PSA can no longer be considered as a classical tumor marker, whose levels are directly associated with the progression of the disease. It has been suggested that the total PSA may even be found to be decreasing, and not increasing, with the elevation of Gleason score[9]. In recent years, although much progress has been made in providing new and efficient markers for screening and diagnosis of prostate cancer, undoubtedly, none of these markers can alone fulfill the diagnostic needs of today in terms of accuracy, precision, and economy. Therefore, the need to find more efficient methods is obvious.

Somatic genetic alterations can be used for diagnostic and prognostic purposes, but the routine methods of sampling are so aggressive, and there is an urgent need for development of less aggressive diagnostic tests. Cell-free DNA (cfDNA) circulating in the blood can originate from apoptosis or necrosis of tumor cells and sunsequent release of DNA contents into the bloodstream[10-12,13], and according to what research has revealed today, its level in the blood circulation increases in cancer cases[14]. The research has also shown that the level of cfDNA in malignant cases of cancer is higher than in benign ones[15]. Following this report, the use of cfDNA, as a predictive marker for prostate cancer, has been evaluated in several studies[16-20]. Nonetheless, there are some controversies among researchers on the exact relationship between the cfDNA levels and the severity of the disease. By investigating the cfDNA levels in patients with prostate cancer as well as healthy subjects and also by looking into its relationship with blood PSA levels, we have tried to find out the diagnostic value of these biomarkers in prostate cancer screening.

## MATERIALS AND METHODS

### Patients

The study was conducted on 76 patients with prostate cancer (50 patients with localized prostate cancer and 26 patients with metastatic prostate cancer) over a period of two years, from January 2014 to November 2015. The patients under study referred to one of the two hospitals, Labbafinejad and Sina, located in Tehran, Iran. After the diagnosis of prostate cancer following digital rectal exam and measurement of PSA serum levels by ELISA method, the patients were referred to be included in the study. Patients entered the study with the following criteria: urinary tract symptoms, positive digital rectal exam (lumps, nodules, the presence of abnormal tissue in the prostate), and serum PSA level above 2.5 ng/ml for individuals between 50 to 65 years old and more than 4 ng/ml for people older than 65 years. The histological degree of tumor for all biopsied samples was determined by the Gleason scoring system[7,21]. In addition, 50 patients with BPH, who had been admitted to the same hospitals, were considered as controls.

### Sampling

After obtaining a written consent approved by the Ethics Committee (No. IR.SBMU.UNRC.1393.2) of the Shahid Beheshti University of Medical Sciences (Tehran, Iran) and with the informed consent of the participants, samples of 10 ml were drawn from the venous blood of all cases. In order to avoid raising the false cfDNA level and other factors important in the peripheral blood of individuals[22-27], sampling was performed before the intervention of any treatment, and before prostate surgery was conducted. Blood samples were transferred to tubes containing the anti-coagulant EDTA (Mediplus, UK). Within three hours after collection and with 10 minutes of centrifugation at 4°C at 2500 ×g, in order to minimize cell lysis and DNA release, blood plasma was isolated. The plasma samples were maintained at -80 °C until use.

### Extraction of cfDNA

cfDNA extraction from plasma was performed using a MagCore HF16 (RBC Bioscience, New Taipei City, Taiwan) and its proprietary kit (Kit number 105), according to the protocol provided by the manufacturer. Quantification and purity of the isolated cfDNA were spectrophotometrically determined at 260 and 280 nm in duplicate using a NanoDrop Spectrometer ND-1000C (Thermo Fisher Scientific, USA). Quantitative analysis of serum PSA levels was performed by ELISA. DNA isolation using the magnetic beads system yielded the highest quantity with the best quality of total plasma DNA.

### Statistical analysis

Statistical analysis of the data was conducted using SPSS version 16 (SPSS Inc., Chicago, IL, USA). To compare the average serum levels of PSA and the plasma concentration of cfDNA between the groups, ANOVA test was performed. In all the cases, the significance level was set at 0.05. The Pearson correlation test was used to examine the relationship between each of the two variables.

## RESULTS

The results of statistical analysis showed that the mean age of the patients with localized prostate cancer to be 63.16 ± 4.50 years (age range 55–70 years), 66.26 ± 4.37 years (age range 55–72 years) for patients with metastatic prostate cancer, 42 ± 5.62 years (age range 54–76 years) for patients with BPH, and 61.00 ± 3.91 years (age range 55–68 years) for normal subjects. Significant differences between different age groups were not observed (*p* = 0.097; Table 1).

**Table 1:** Demographic data of patients under the study

Characteristics	Case group (n = 76)		Control group (n = 60)		*P* value
	
	Patients with clinically localized PCA (n = 50)	Patients with metastatic PCA (n = 26)	BPH (n = 50)	Normal (n = 10)	
Age (years) Mean ± SD	63.16 ± 4.50	66.26 ± 4.37	61.42 ± 5.62	61.00 ± 3.91	0.097
Range	55-70	55-72	54-76	55-68	
≤65 n (%)	34 (68.00)	9 (34.61)	37 (74.00)	8 (80.00)	
>65 n (%)	16 (32.00)	17 (65.39)	13 (26.00)	2 (20.00)	
Gleason scores					0.000
≤7 n (%)	49 (98.00)	17 (65.38)	-	-	
>7 n (%)	1 (2.00)	9 (34.62)	-	-	
Serum PSA (ng/mL)					0.001
Mean level ± SD	18.48 ± 2.28	28.20 ± 3.46	7.93 ± 3.71	1.42 ± 0.72	
Range	4.50-112.0	5.90-121.0	1.27-20.5	0.50-2.50	
cfDNA level (ng/µl)					0.000
Mean level ± SD	15.04 ± 3.21	19.62 ± 4.82	9.51 ± 2.13	7.8 ± 1.29	
Range	10.5-25.10	12.5-31.30	6.30-14.5	6-10	

Clinical studies of individuals in the case group using the Gleason scale indicated that the Gleason score was less than or equal to 7 in 49 patients (98%) with localized prostate cancer patients and 17 (65.38%) patients with metastatic prostate cancer. The Gleason score was higher than 7 in one patient (2%) in the first group and 9 patients (34.62%) in the second group (Table 1).

Measurement of the serum levels of PSA by ELISA method showed that the mean serum levels of PSA were as follows: 28.20 ± 3.46 ng/ml (range 5.90–121.0 ng/ml, in patients with metastatic prostate cancer), 18.48 ± 2.28 ng/ml (range 4.50–112.0 ng/ml, in patients with localized prostate cancer), 7.93 ± 3.71 ng/ml (range 1.27–20.5 ng/ml, in patients with BPH), and 1.42 ± 0.72 ng/ml (range 0.5–2.5 ng/ml, in healthy subjects). Data comparison demonstrated a significant difference between the mean serum levels of PSA in the control and case groups (*p* = 0.001; Table 1).

Mean plasma concentration of cfDNA in patients with metastatic prostate cancer was 19.62 ± 4.82 ng/μl (range 12.5–31.30 ng/μl) and in patients with localized prostate cancer was 15.04 ± 3.21 ng/μl (range 10.5– 25.10 ng/μl), whereas it was 9.51 ± 2.13 ng/μl (range 6.50–14.5 ng/μl) in patients with BPH and 7.8 ± 1.29 ng/μl (range 6–10 ng/μl) in normal subjects. There was a significant difference between the groups in terms of the mean plasma concentration of cfDNA (*p* < 0.0001; Table 1).

To investigate the relationship of cfDNA level among the groups of patients with metastatic prostate cancer and localized prostate cancer and also that with BPH, ANOVA test was used. Given F = 107.312 and *p* < 0.0001, there were statistically significant differences between all the groups. It is clear that there was a significant diffidence between metastatic prostate cancer and localized prostate cancer cases and even in BPH cases with both groups of cancer (metastatic and localized).

The Pearson correlation test, which was used to examine the relationship between cfDNA level and age, displayed a weak correlation between these two variables (*p* < 0.0001 and r = 0.306). The results also showed that with increasing age, the cfDNA in plasma levels increased (Fig. 2A).

**Fig. 1:**
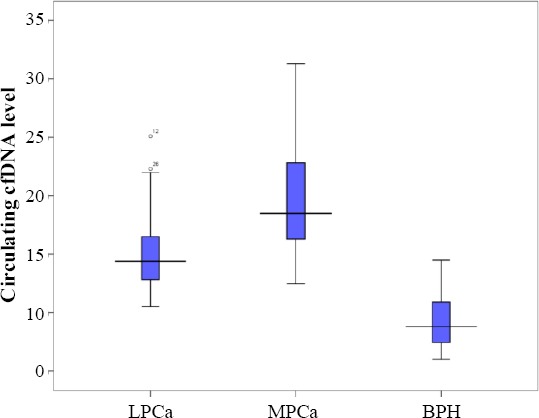
Box plot comparison of plasma DNA concentrations in groups. Numbers show cfDNA level as ng/ml. LPCa, localized prostate cancer; MPCa, metastatic prostate cancer; BPH, benign prostatic hyperplasia

**Fig. 2:**
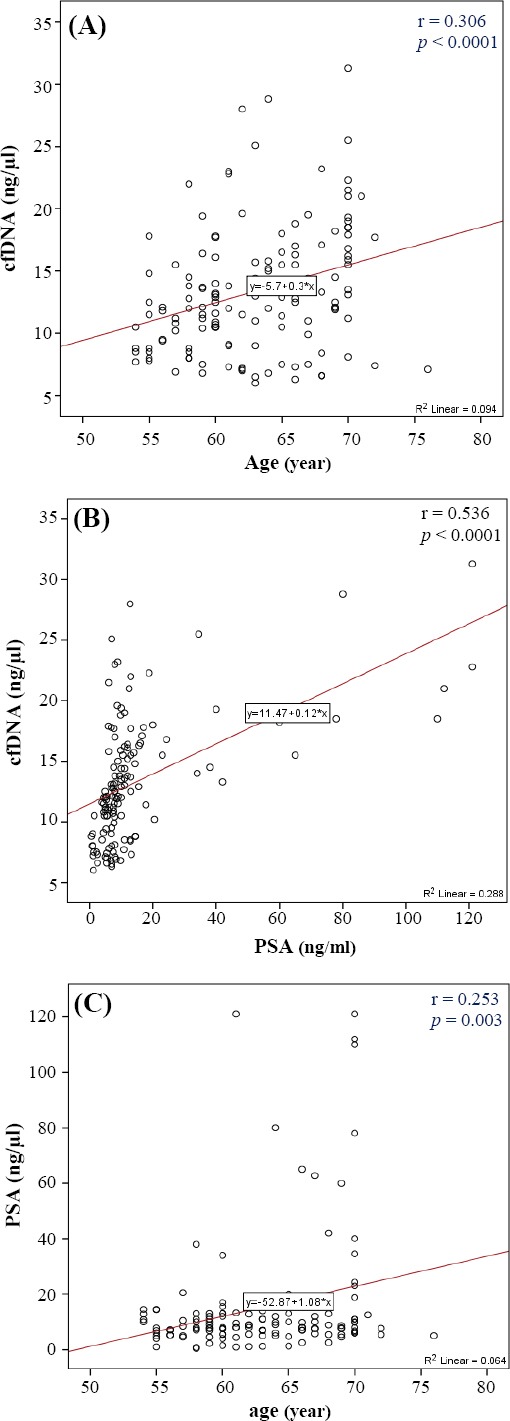
Correlation of cfDNA level with age and PSA levels and the correlation of PSA levels with age. The scatter diagrams show the Pearson correlation coefficient in A (cfDNA level and age), B (cfDNA level and PSA level), and C (PSA level and age).

The relationship between the variables, cfDNA levels, and PSA levels, using Pearson correlation test, showed that there is a correlation between cfDNA and PSA (*p* < 0.0001 and r = 0.536). The correlation intensity obtained was moderate. The results also indicated that with increasing PSA levels, cfDNA in plasma levels also increased (Fig. 2B).

The Pearson correlation test examined the relationship between age and PSA level and suggested a weak correlation between the two variables (*p* = 0.003 and r = 0.253). The results also showed an increase in PSA levels with increasing age (Fig. 2C).

## DISCUSSION

To date, many applications have been proposed for cfDNA, particularly its use in identifying somatic changes in cases where there is no possibility of biopsy. In addition, this exact molecule can be a valuable source of the DNA tumor in cases where the exact origin of primary lesions is not clear. In addition, cfDNA can be used as a very important screening marker in population-based studies[10]. While investigating the cfDNA levels of patients with prostate cancer and its comparison with the cfDNA levels of healthy individuals, this study tried to evaluate this marker for use in prostate cancer screening.

The first observations led to the conclusion that cfDNA is caused by tumor tissue, suggesting that there are some mutations in the proto-oncogenes and tumor suppressors, such as KRAS2 and TP53 in the tumor tissue and in cfDNA. Also, as confirmation of these observations, cfDNA in cancer patients has biophysical properties similar to tumor cells[28]. However, the ease of collection and reproducibility of sampling introduce cfDNA as a suitable marker for tumor tracking during treatment[29]. On the other hand, Diehl *et al*.[30] have suggested that the ratio of tumor cfDNA to total cfDNA depends on various factors, such as tumor size, cancer type, disease stage, and the location of the tumor. In the present study, there were significant differences between cfDNA levels in the blood of patients with localized and metastatic prostate cancers. These levels were significantly higher in the second group of the patients (*p* < 0.0001).

In two previous studies, Stroun and Anker[31] and Anker *et al*.[32] suggested that the enzyme activity of DNase 1 and DNase 2 in healthy people damages DNA in circulating blood. However, little activity of these enzymes has been observed during malignancy. The reason is due to the inhibitors of enzyme activity in cancers and can be explained by the high levels of cfDNA. In this study, there was a significant difference between the case and control group in the plasma cfDNA level (*p* < 0.0001). Barry *et al*.[33] showed that the high level of cfDNA concentration is because of decreasing DNAse activity.

Until now, a definitive and an approved mechanism for the process of entering healthy cells into the bloodstream has not been recommended. A previous study showed the high level of cfDNA in PCa plasma patients compared to BPH[34]. The only proposed hypothetical mechanisms for this phenomenon include apoptosis, necrosis, and release of healthy cells into the bloodstream, followed by destruction of the cells and release of their DNA contents into the bloodstream[10].

There have been cases of healthy cells being isolated from the bloodstream of patients with prostate, liver, and breast cancers[34]. There was a good agreement between the DNA hypermethylation patterns of circulating tumor cells and cfDNA in prostate cancer[35,36]. The destruction of tumor cells in the bloodstream can be considered as a reason for the presence of large pieces of cfDNA in the blood. In a study by Barry *et al*.[33], an eightfold increase was observed in the median levels of cfDNA, as a marker, in lung cancer cases in comparison to healthy patients. Following varoius reports[34-37], the use of cfDNA, as a marker for prostate cancer, has also been evaluated in other studies[16,17]. One of the studies showed that there is no difference between the levels of cfDNA in patients with localized prostate cancer and BPH; however, the levels of this marker indicated a significant increase in prostate cancer metastasis[18]. In contrast to these findings, there was a significant difference between the levels of cfDNA in patients with localized prostate cancer and BPH in the present study (*p* < 0.0001).

There have been successful efforts to differentiate between patients with prostate cancer and healthy people using cfDNA[38]. The possibility of using cfDNA in diagnosis has been approved as a result of all these efforts[16-20,38]. According to the findings of, our study, it seems that there are significant differences in cfDNA levels in patients with metastatic prostate cancer and patients with localized prostate cancer. The significant difference of cfDNA level among all groups in the present study indicate the importance of using these biomarkers in non-invasive diagnostic procedures. We showed older men have higher PSA and cfDNA in their blood. It means that there is a correlation between age, plasma DNA, and PSA. Thus, cfDNA can be used as a suitable substitute for biopsies in the future with further developments in the field of cancer-screening tests and tests that require DNA samples of cancer cells. Probably, cfDNA level assessment together with PSA can be complementary tests for early screening. Gordian *et al*.[38] have reported that cfDNA level increases the specificity of PSA test, especially in early prostate cancer detection.

As a conclusion, cfDNA can be used as a non-invasive and a promising novel biomarker; therefore, it could be a robust tool for prognosis and diagnosis goals. In addition, detection of genetic alterations in cfDNA such as loss of heterozigocity along with cfDNA level in prostate cancer patients may help in early diagnosis[39]. However, further studies, on a large scale of population, may be required to validate these data.
